# Should All Minimal Access Surgery Be Robot-Assisted? A Systematic Review into the Musculoskeletal and Cognitive Demands of Laparoscopic and Robot-Assisted Laparoscopic Surgery

**DOI:** 10.1007/s11605-022-05319-8

**Published:** 2022-04-14

**Authors:** Abdul Shugaba, Joel E. Lambert, Theodoros M. Bampouras, Helen E. Nuttall, Christopher J. Gaffney, Daren A. Subar

**Affiliations:** 1 Lancaster Medical School, Lancaster University, Lancaster, LA1 4YG UK; 2 Royal Blackburn Hospital, East Lancashire NHS Hospitals Trust, Blackburn, BB2 3HH UK; 3 Department of Psychology, Lancaster University, Lancaster, LA1 4YG UK

**Keywords:** Posture, Ergonomics, Fatigue, Cognitive, Mental

## Abstract

**Background:**

Surgeons are among the most at risk of work-related musculoskeletal health decline because of the physical demands of surgery, which is also associated with cognitive fatigue. Minimally invasive surgery offers excellent benefits to patients but the impact of robotic or laparoscopic surgery on surgeon well-being is less well understood. This work examined the musculoskeletal and cognitive demands of robot-assisted versus standard laparoscopic surgery.

**Methods:**

Medline, Embase and Cochrane databases were systematically searched for ‘Muscle strain’ AND ‘musculoskeletal fatigue’ AND ‘occupational diseases’ OR ‘cognitive fatigue’ AND ‘mental fatigue’ OR ‘standard laparoscopic surgery’ AND ‘robot-assisted laparoscopic surgery’. Primary outcomes measured were electromyographic (EMG) activity for musculoskeletal fatigue and questionnaires (NASA-TLX, SMEQ, or Borg CR-10) for cognitive fatigue. A systematic review was conducted in accordance with the Synthesis Without Meta-analysis (SWiM) Guidelines. The study was preregistered on Prospero ID: *CRD42020184881.*

**Results:**

Two hundred and ninety-eight original titles were identified. Ten studies that were all observational studies were included in the systematic review. EMG activity was consistently lower in robotic than in laparoscopic surgery in the erector spinae and flexor digitorum muscles but higher in the trapezius muscle. This was associated with significantly lower cognitive load in robotic than laparoscopic surgery in 7 of 10 studies.

**Conclusions:**

Evidence suggests a reduction in musculoskeletal demands during robotic surgery in muscles excluding the trapezius, and this is associated with most studies reporting a reduced cognitive load. Robotic surgery appears to have less negative cognitive and musculoskeletal impact on surgeons compared to laparoscopic surgery.

**Supplementary Information:**

The online version contains supplementary material available at 10.1007/s11605-022-05319-8.

## Introduction

Surgeons are amongst the most at risk of work-related musculoskeletal decline,^1^ with a high prevalence of work-related musculoskeletal injuries: degenerative spinal disease (17%), rotator cuff pathology (18%) and degenerative lumbar spine disease (19%).^2^ Additionally, they experience a high rate of work-related musculoskeletal pain predominantly affecting the neck, arm, shoulder and back.^3–5^ These problems relate to the nature of their jobs requiring them to maintain certain non-ergonomic postures whilst operating (mostly for long periods), with a cumulative effect over time. Maintaining unnatural postures for prolonged surgery periods can result in muscle fatigue. As skeletal muscle fatigues during surgery, the central nervous system attempts to compensate by activating a greater number of motor neurones or by increasing their discharge rate ^17^. As a consequence, surgeons feel they are exerting more effort to maintain a given muscle contraction.^6^

In addition to musculoskeletal limitations, extended working patterns in surgeons also lead to cognitive fatigue. Surgeons are required to engage in numerous surgical processes requiring sustained attention for long periods, often following long working hours or sub-optimal sleep resulting in cognitive fatigue.^7^ Studies in the workplace have clearly established a relationship between cognitive fatigue and impaired performance, including slower reaction times,^8^ reductions in concentration^9^ and impaired memory and information processing.^10^ This has extensively been researched amongst pilots^11^ and train operators,^12^ clearly demonstrating that cognitive fatigue is associated with decreased overall performance and safety. This has vital consequences, especially in professions which require a very low margin of error to maintain safety. Even studies amongst drivers revealed that cognitive fatigue accounted for 12% of car crashes and 10% of near-misses.^12,13^ Amongst surgeons, level one evidence is lacking but the impact of cognitive fatigue and impaired performance on patients could be critical. Indeed, whilst there is significant heterogeneity in the literature, several studies have shown fatigue can result in increased surgical errors and adverse patient outcomes.^7^

Whilst this increased utilisation of predominantly standard laparoscopic techniques, it provides favourable patient outcomes; it inadvertently increases musculoskeletal demands (MSD) experienced by surgeons due to the limited freedom of movement, limitations in instrument design, longer operating time (in some procedures) and poor positioning of the operating room table and monitors.^14^ Increased workplace MSD and musculoskeletal symptoms increase total fatigue and lower both concentration and focus,^15,16^ thereby decreasing the accuracy of performing cognitive tasks.^17,18^

Traditional open surgery is associated with increased musculoskeletal pain and discomfort, predominantly attributed to non-ergonomic postures adopted by surgeons^19,20^; therefore, to mitigate these problems, the modern technology of robot-assisted laparoscopic surgery (RALS) may help reduce musculoskeletal problems in surgeons when compared to standard laparoscopic surgery (LS). RALS offers steadier wrist movements with a reduced fulcrum effect, the surgeon is sat on a console with an arm rest assuming a natural working axis, and the console provides a 3-dimensional image of the operating field, which improves stereoscopic depth perception.^21^ In comparison, surgeons are mostly standing to perform LS procedures and must remain scrubbed donning the additional Personal Protective Equipment (PPE) required but remain unscrubbed during RALS. The symptoms of pain or discomfort reported by surgeons performing LS procedures predominantly affect the back, neck, lower extremities and shoulders with a prevalence of 73–90%.^22–24^ This can potentially be improved with RALS.

To our knowledge, no systematic review exists that has directly compared RALS to standard LS with respect to musculoskeletal and cognitive implications of these two types of surgery. A better understanding of the similarities and differences with regard to musculoskeletal and cognitive impact on the surgeons will have significant impact on surgeons and patients alike, with the potential to provide essential evidence to direct the course of future surgical training and enhance health outcomes. This paper therefore aimed to comprehensively review the available scientific literature and report on the musculoskeletal demands in surgeons performing RALS as compared to LS, and the associated cognitive fatigue.

## Methods

A qualitative systematic review was conducted in accordance with the Systematic Review Without Meta-Analysis (SWiM) Guidelines,^25^ as a meta-analysis was deemed not appropriate due to the heterogeneity in study designs and their reported outcomes.

### Literature Search Strategy and Study Selection

The literature search was developed around the concepts of ergonomics, minimally invasive surgery and surgeon fatigue. Using Boolean operators to combine different ‘MeSH’ and ‘non-MeSH’ keywords, a systematic literature search was conducted in Medline, Embase and Cochrane databases with no start date but including papers published up until 31 October 2020. The search terms used were ‘Muscle strain’ AND ‘musculoskeletal fatigue’ AND ‘occupational diseases’ OR ‘cognitive fatigue’ AND ‘mental fatigue’ OR ‘Standard laparoscopic surgery’ AND ‘robot-assisted laparoscopic surgery’. Appendix 1 shows a typical search strategy employed in a database.

Studies that met the following criteria were included in the systematic review: (1) published as a full text manuscript; (2) not a protocol or review manuscript; (3) studies involving surgeons performing elective standard laparoscopic and robot-assisted laparoscopic surgery or simulated laparoscopic and robot-assisted procedures (4) objectively or subjectively report on musculoskeletal and/or cognitive demands of surgery. Only English language papers were reviewed, with no restrictions applied on the surgical specialty, procedures studied or study design.

### Data Extraction

Outcomes recorded for muscular and cognitive fatigue were objective physiological parameters associated with muscular or cognitive fatigue, as well as more subjective measures using validated questionnaires of physical symptoms, pain or discomfort, scales of perceived discomfort, Borg CR-10 scale^26^ and national aeronautics and space administration task load index (NASA-TLX).^27^

The outcome used for fatigue was muscle fibre recruitment assessed via the use of electromyography (EMG).^28^ When a contracting muscle fatigues, it attempts to recruit more muscle fibres or alters the firing rate. These changes indicate the muscle’s decreasing ability to maintain the required force generation and have been used to assess fatigue in surgeons. Musculoskeletal fatigue was determined using surface electromyography (EMG) data. Where reported, the root mean square (RMS) value represents the square root of the average power of the EMG signal for a given time. The cumulative muscle workload (CMW) over the period of performance time can also be calculated using a time integral of the data collection period.^29^

Cognitive fatigue was determined using heart rate parameters derived by registering participants’ heart rates throughout experiments or at specific times using an ambulatory heart rate recorder, calculating the heart rate average and mean square of successive differences between consecutive heartbeats.^30^ Skin conductance was also utilised, where a single electrode was placed on an active site with a reference electrode at a relatively inactive site and a measured potential (which is usually negative) is easily recorded as a complex wave form. Higher values are indicative of physiological arousal due to increased sympathetic autonomic nervous activity. This is sensitive to physiological reactivity among other factors, such as respiration and cognitive effort. Metrics that can differentiate between increased cognitive loads can also be generated from this.^31^

Pain or scales of perceived discomfort were assessed using validated questionnaires using a Likert scale to rate perceived symptoms giving different scores which are then summed up to give a cumulative score.

The individual rating of perceived exertion was assessed using the Borg CR-10 scale during physical work, rating their exertion on the scale of 1 to 10 during the activity, combining all sensations and feelings of physical stress and fatigue. The NASA-TLX is a tool for assessing subjective cognitive load which incorporates measures from six dimensions (mental demand, physical demand, temporal demand, effort, performance and frustration level) which are rated within a 100-point range and a sum is then calculated.

Effect sizes were converted into a common metric of *p* values or percentages before analysis. In addition to the primary outcomes, other data extracted also included study author, year published, study design, surgeon demographics and hand dominance.

### Data Analysis

A qualitative systematic review was performed of the reported outcomes comparing RALS and LS. When reviewing the results of previous studies, we defined statistical significance as *p* < 0.05.

## Results

### Study Selection

**Fig. 1:**
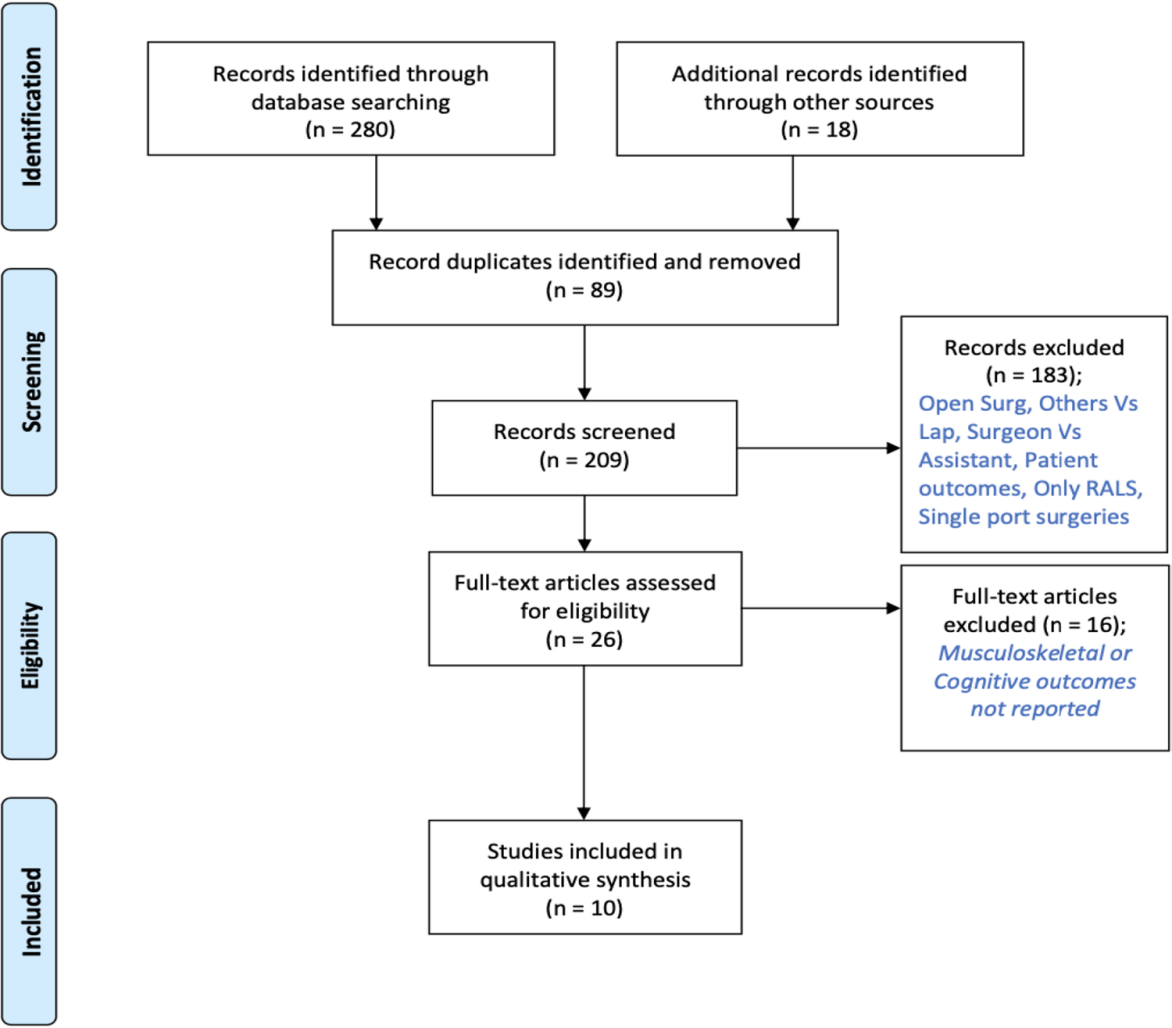
PRISMA flow diagram of studies included in the systematic review

A systematic search of the available literature returned 298 articles. After eliminating duplicates, 209 articles remained. When irrelevant titles and abstracts were screened out based on the inclusion criteria, 26 articles were preliminarily included. After scrutinising the retrieved full texts of these articles, 10 articles remained (Fig. 1) which met the criteria to be included in the review. The study selection process was verified by a second reviewer (J.L.) scrutinising 10% of the selected studies.

### Study Characteristics

The quality of each study was critically appraised using the grading recommendations assessment, development and evaluation (GRADE) framework^32^ (Table 1). All the studies were considered to at least be of ‘fair’ quality. Of the ten articles included in this systematic review, none were randomised controlled trials, and all were observational studies (Table 2). All studies examined both the musculoskeletal demands and the cognitive demands of surgery.

**Table 1:** GRADE evidence profile

Comparison of the musculoskeletal and cognitive demands of performing robot-assisted (RALS) versus standard laparoscopic (LS) surgery									
**Population:** Surgeons performing Minimal access surgeries **Setting:** Operating theatre and surgical simulation environments **Intervention:** Robot-assisted laparoscopic surgery **Comparison:** Standard laparoscopic surgery									
**Certainty assessment**							**Impact**	**Certainty**	**Importance**
**No. of studies**	**Study design**	**Risk of bias**	**Inconsistency**	**Indirectness**	**Imprecision**	**Other considerations**			
Musculoskeletal (MSK) fatigue									
10 ^35,38–46^	Observational studies	Not serious	Not serious	Serious ^a^	Serious ^b^	None	Studies reported increased physical demand and MSK fatigue involving biceps, triceps, deltoid and erector spinae with LS. 3 studies showed greater trapezius strain in RALS, especially in surgeons with limited MIS experience	⨁◯◯◯ Very low	Critical
Cognitive fatigue									
10 ^35,38–46^	Observational studies	Not serious	Not serious	Serious ^a^	Serious ^b^	None	Greater cognitive demands were reported with LS evidenced by high mental demand scores, Heart rates and skin conductance values	⨁◯◯◯ Very low	Critical

^a^Differences in outcome measures and variability of study populations

^b^No confidence intervals reported but with low absolute numbers of participants and events

**Table 2:** Summary of reviewed studies

Study and year	Study design	Type of procedure compared	Population	MSK fatigue measure	Outcome	Cognitive fatigue measure	Outcome
Berguer R et al. (2005)^36^	Observational, prospective	Simulated tasks	10	%MVCrms	-Less EMG activation in Thenar Muscles using RALS, *p: 0.02* -No difference in deltoid muscle	Skin conductance, perceived discomfort	-Lower Skin conductance values in RALS, *p:0.056* -Perceived discomfort Lower in LS amongst experienced surgeons for 1st task and no difference for 2nd task
Van Der Schatte Olivier Rv et al. (2009)^39^	Observational, prospective	Simulated tasks	16	LED	Higher discomfort scores in LS, *p: 0.003*	-RMSSD, PEP and HRA -SMEQ	- Lower values of physiological parameters with RALS, *p: 0.01, 0.004 and 0.0001* respectively - Lower mental effort associated with RALS, *p: 0.001*
Stefanidis D et al. (2011)^40^	Observational, prospective	Simulated tasks	117	NASA-TLX	LS more physically demanding, *p:* < *0.001*	NASA-TLX	Similar mental demands in both RALS and LS, *p:* < *0.05*
Lee G et al. (2014)^32^	Observational, prospective	Simulated tasks	13	CMW (EMG)	All muscle groups apart from the trapezius showed lower values in RALS, *p:* < *0.05*	NASA-TLX	Lower cognitive load with RALS, *p:* < *0.05*
Sánchez A et al. (2017)^41^	Observational, prospective	Simulated procedure	14	LED	Lower physical disturbance in RALS, *p:0.04*	SMEQ	Lower Mental effort in RALS, *p:0.001*
Rodriguez JGZ et al. (2018)^37^	Observational, prospective	Simulated tasks	31	%MVC	Lower muscle activation except in trapezius in RALS, *p:* < *0.01*	NASA-TLX	Lower cognitive demand scores in RALS, *p:* < *0.01*
Hubert N et al. (2013)^38^	Observational, prospective	Simulated procedure	11	%MVC/NASA-TLX	Lower physical workload scores in RALS*, p:* < *0.05*	-HR -NASA-TLX and Borg CR-10	-Lower average HR in RALS, *p*: < 0.01 -No difference in RALS and LS scores, *p:* < *0.05*
Tarr ME et al. (2014)^43^	Observational, prospective	Live procedures	16	BPD survey	Lower discomfort scores in RALS, *p:0.03*	NASA-TLX	No difference between RALS and LS
Armijo PR et al. (2018)^42^	Observational, prospective	Live procedures	16	%MVCrms	Less activation with RALS, *p:0.003*	PFH-12	No difference in self-reported fatigue, *p:0.869*
Mendes V et al. (2019)^44^	Observational, prospective	Live procedures	24	Borg	Lower physical discomfort scores in RALS, *p:* < *0.05*	NASA-TLX	Lower load scores in RALS, *p:* < *0.05*

*MSK* musculoskeletal, *%MVCrms* root mean square of maximal voluntary contraction, *EMG* electromyography, *RALS* robot-assisted laparoscopic surgery, *LS* standard laparoscopic surgery, *LED* Local Experienced Discomforts scale, *RMSSD* root mean square of successive differences between consecutive heartbeats, *PEP* pre-ejection period, *HRA* heart rate average, *SMEQ* Subjective Mental Effort Questionnaire, *NASA-TLX* National Aeronautics and Space Administration total load index score, *CMW* cumulative muscular workload, *Borg CR10* Borg rating of perceived exertion, *BPD* body part discomfort, *PFH-12* Piper Fatigue Scale-12

### Musculoskeletal Demands of Laparoscopic Versus Robot-Assisted Minimally Invasive Surgery

Robotic systems are designed to provide surgeons with access to physiological structures in otherwise difficult to reach areas, whilst also providing finer endowristed movements to simplify MIS surgical procedures. The studies in this review involved live and simulated procedures, with the simulated procedures replicating ‘real-world’ tasks and challenges.

#### Data from Electromyography

Berguer and Smith^33^ utilised objective outcomes to report lower musculoskeletal demands in 10 surgeons in a simulated type of surgery study. Participants each performed in random order, A Pin Move task (PIN), picking up a poster pin standing on its head in a circle and attempting to set it down standing on its head in another circle; and a suture task (SUT), involving driving a suture needle through a surgical glove finger and tying three knots (one surgeon’s knot plus two squared throws). Using the RALS technique, significantly lower thumb muscle activity was observed performing the SUT task and although more abduction was required to perform the PIN task, muscle activity values in the deltoid were not correspondingly higher.

Similarly, Lee et al.^29^ and Rodriguez et al.^34^ objectively reported less physical demands associated with RALS. Rodriguez et al.^34^ described higher muscle activity in bilateral biceps, triceps and deltoid muscle groups when Fundamentals of Laparoscopic Surgery (FLS) tasks (peg transfer, pattern cutting and intracorporeal suturing) were performed using standard laparoscopy across the study groups; novices, surgical experience in LS and surgical experience in RALS. Additionally, they also reported higher muscle activity in the right trapezius across the groups with different surgical expertise when they performed peg transfer and paper cutting using the robotic platform but not for intracorporeal suturing, with these being statistically significant (novices: *p* = 0.04 and *p* < 0.01, LS experts: *p* = 0.04 and *p* = 0.04 *and* RALS experts: *p* = 0.04 and *p* = 0.01 respectively). Lee et al.^29^ reported similar findings when six more complex simulated tasks which included simulated para-oesophageal hernia repair, simulated bowel anastomosis, tension running suturing, FLS circle cutting, curved wire ring transfer and FLS pegboard transfer were performed. They reported significantly higher cumulative muscular workload (CMW) of the biceps and the flexor carpi ulnaris with laparoscopy (both *p* < 0.05) compared to RALS but a higher CMW from the trapezius during robotic surgery performance (*p* < 0.05). Investigating this further, they reported that only the novice and expert laparoscopic groups exhibited higher trapezius activation (*p* = 0.052 and *p* = 0.081 respectively), whilst the robotic experts displayed similar activation levels in both approaches. In addition, there was evidence (*p* = 0.06) of higher CMW of the thenar compartment with robotic surgery than with laparoscopy, due to increased usage of finer finger movements with RALS. 

The Armijo et al.^35^ study involved 16 surgeons from different specialities, predominantly right-handed with equal gender distribution performing live procedures (18 LS and 10 RALS) within fields in which they were deemed competent. Although the authors reported greater muscle activation across the upper trapezius (*p* = 0.190), anterior deltoid (*p* = 0.066) and flexor carpi ulnaris (*p* = 0.170) in the robot group using %MVC, no difference in muscle fatigue in the same muscle groups was noted. However, they observed a significant increase in fatigue in the extensor digitorum of the LS group (*p* < 0.001).

#### Data from Validated Questionnaires of Musculoskeletal Demands

Van der Schatte Olivier et al.^36^ also studied novices: surgically inexperienced students, performing rope passing, needle capping and bead dropping. The physical demands experienced when these tasks were performed laparoscopically were significantly greater as indicated by high Subjective Mental Effort Questionnaire (SMEQ) and Local Experienced Discomfort scale (LED) scores (*p* = 0.001 and *p* = 0.003, respectively).

Stefanidis et al.^37^ studied a cross section of 117 surgeons attending an academic conference using the NASA-TLX’s different domains to capture the physical demands they experienced whilst performing simulated intracorporeal suturing. Most participants achieved higher suturing scores with the laparoscopic technique but reported significantly more physical demand scores (*p* < 0.001) compared to those performed with the robotic platform and subjectively favoured the robot as their method of choice.

Using similar tools as Van der Schatte Olivier et al.,^36^ a study by Sánchez et al.^38^ surveyed 14 surgeons experienced in standard laparoscopic surgery after they had performed a simulated hernia repair using both LS and RALS. They reported predominantly higher physical demands (high LED scores, *p* = 0.006) in the surgeons’ dominant upper limb when the task was performed using the laparoscopic approach.

Mendes et al.^39^ categorised their participants based on experience, similar to how it was done by authors of some simulated studies, into young surgeons (< 7 years in practice, 45%) and experienced surgeons (> 7 years in practice, 55%). The study population comprised of surgeons from three specialties, and they cumulatively performed a total of 82 laparoscopic and 88 robotic procedures with a mean duration of 119 min and 157 min, respectively. Using the Borg CR-10 scale scores, the authors reported significantly greater physical discomfort and pain in surgeons performing laparoscopic procedures with no significant difference in these outcomes based on experience of the surgeons. The exception was significant back pain reported after the 150th minute of robotic procedures in experienced surgeons (*p* < 0.01). Using the NASA-TLX scores, experienced surgeons had a feeling of better performance at the end of LS compared to RALS (*p* = 0.02) but also expressed more physical demands performing LS (*p* = 0.03).

Tarr et al.^40^ conducted a pilot study in a population of predominantly female (75%) surgeons performing 53 laparoscopic and 33 robotic sacrocolpopexy cases and reported no statistically significant differences in both physical (body part discomfort (BPD) & NASA-TLX scores) and cognitive loads (NASA-TLX) observed (*p* = 0.66 and *p* < 0.05, respectively). After dichotomising BPD scores, surgeons were noted to have experienced pain in all body parts except their arms, across both study groups. Additionally, the robotic approach was associated with increased lower neck/shoulder and back discomfort scores compared to the laparoscopic approach.

#### Data Derived from Mixed-Method Approaches

The study by Hubert et al.^41^ simulated live surgical procedures in experimental animals whilst monitoring 11 surgeons perform a total of 18 laparoscopic and 16 robotic procedures. Unlike the studies by Lee et al.^29^ and Rodriguez et al.^34^ using EMG data, the authors reported higher RMS (*p* < 0.05) for the erector spinae, trapezius and the flexor digitorum on both the right and left muscle groups, when procedures were performed laparoscopically, and the values also increased in both trapezius muscles at the end of the procedures. During the laparoscopic procedures, the authors also reported high NASA-TLX and Borg CR-10 scores for all body areas (*p* < 0.05 and *p* < 0.001, respectively) suggesting more physical demands, with the greatest strain in the shoulders, neck and back.

### The Associated Cognitive Demands of Laparoscopic Versus Robot-Assisted Minimally Invasive Surgery

Berguer and Smith^33^ utilised skin conductance values to observe surgeons’ cognitive fatigue reporting lower cognitive load with RALS technique in both PIN and SUT tasks, though not statistically significant (*p* < 0.056).

To measure cognitive demands, Rodriguez et al.^34^ reported high NASA-TLX scores in temporal demand in both novices and experts in laparoscopic surgery, when they completed FLS tasks using the laparoscopic platform (*p* = 0.02 and *p* = 0.02). No change in temporal demands was observed in surgeons who were experts in robotic surgery when they performed procedures using RALS or LS. Lee et al.^29^ also found significantly higher NASA-TLX scores relating to temporal demand, and frustration with LS than with RALS (*p* < 0.05). This was especially evident in novices and experts in robotic surgery when they performed FLS and even more complex simulated tasks.

Another study by Hubert et al.^41^ subjectively analysed cognitive fatigue using NASA-TLX scores and observed no difference between LS and RALS. However, when cognitive fatigue was assessed using mean heart rate vales and heart rate variability as objective measures, they noted both parameters to be significantly higher in the laparoscopic group (both *p* < 0.01).

Van der Schatte Olivier et al.^36^ also utilised heart rate parameters as objective physiological markers to highlight the increased cognitive demands participants experienced when tasks of rope passing, needle capping and bead dropping were performed laparoscopically. They reported a higher heart rate average in the LS group of 90.5 beats/min compared to 79.9 beats/min in the RALS group and a corresponding higher root mean square of successive differences between consecutive heartbeats of 31.7 ms in the LS group compared to 22.3 ms in the RALS group (*p* = 0.01 and *p* = 0.0001). This finding was further strengthened by the reporting of high SMEQ scores, which were similar to the findings reported by Sánchez et al.^38^ (high SMEQ score, *p* = 0.001).

In a study by Stefanidis et al.,^37^ in which only 10% of surgeons with prior RALS experience were surveyed, surgeons reported numerically similar NASA-TLX scores of cognitive demand on the robotic platform and the laparoscopic, and this was not statistically significant. Armijo et al.^35^ did not reveal any difference in global self-reported fatigue levels (Piper Fatigue Scale-12 (PFH-12)) between the two surgical approaches. Further scrutiny of this revealed high scores in the behaviour subscale domain being reported for both approaches, and this related to increased cognitive exhaustion.

The study by Mendes et al.^39^ observed that young surgeons experienced more cognitive demands (*p* = 0.02) at the end of RALS. Interestingly, the surgeon who performed the most procedures during the study expressed significantly less cognitive fatigue at the end of RALS.

## Discussion

Minimally invasive surgery improves post-operative pain and patient recovery times, and reduces length of hospital stay.^42–44^ However, historically MIS procedures are predominantly performed using the laparoscopic approach, with reported increased incidence of muscle strain affecting the back, neck, lower extremities and shoulders in surgeons.^22–24^ With the robotic console, surgeons use a chair and have an arm rest for support, eliminating any additional lower limb physical demands unlike when surgeons are mostly standing to perform laparoscopic procedures. This has been demonstrated in studies showing lower muscle activity in the tibialis anterior, medial gastrocnemius, vastus medialis and biceps femoris when performing RALS^45^ and also reduced physical demands on the knee/ankle/foot when performing RALS.^40^ As such, studies have focused on the comparative differences in the upper limb, trunk and neck muscles, when procedures are performed using RALS or LS.

The data presented in this review predominantly involved studies conducted in simulated^40^ as opposed to real-life procedures,^40^ which is a representation of the lacking data in the field of ergonomics relating to surgeons’ use of new technologies. The evidence suggests there is a reduction in musculoskeletal demands of RALS in both simulated and real-life procedures. Similarly, reduced cognitive fatigue was noted with RALS in simulated settings; however, the limited data in real-life procedures suggests no difference.

Overall, this presents the possibility that the robotic approach to minimally invasive surgery has an advantage over the laparoscopic approach. Hence, the data reviewed here suggest that RALS could be the optimal choice with respect to surgeons’ musculoskeletal health, compared to LS. Despite the potential musculoskeletal and cognitive benefits offered by RALS, the theatre and supply costs of robotic surgical systems significantly limits the rate of adoption in surgical settings, especially in low resource settings.^46^

### Musculoskeletal Demands Are Reduced When Performing Robot-Assisted Minimally Invasive Surgery

When fatigue was measured objectively using EMG, there was a consistent increase in musculoskeletal fatigue using a laparoscopic technique including the biceps brachii, triceps brachii, deltoid, trapezius and erector spinae.^29,33–35,41^ Furthermore, Berguer et al.^33^ noted there was reduced fatigue of the thenar muscles when using RALS, which suggests the enhanced grasp provided by the robotic system protects against handgrip fatigue. Studies that utilised subjective measures of musculoskeletal fatigue also showed increased fatigue in the muscles of the upper limbs and back, and importantly, an increased global physical demand with LS.^36–39^

Some studies noted there was an increase in trapezius muscle fatigue when the procedures were performed using RALS,^29,34,35^ but this impact appears confined to this muscle. This can be attributed to the posture that surgeons assume on the robotic console; elbows/forearm rested on the arm-support and assuming a forward-leaning attitude resting their forehead in the viewing cart. This neutralises the arm and shoulders but consequently puts the neck under more strain. Interestingly, the physical strain on the trapezius appears to be modulated by surgeon experience. Indeed, some studies have showed greater (+ 43%^29,34^) trapezius strain amongst surgeons with minimal experience performing RALS (MIS novices) compared to experts. Others^29,34^ have shown a reduction in left trapezius activation with greater expertise but an increase in right trapezius strain, which may suggest that with experience, a particular posture is adapted which puts unique (rightward) strain on the trapezius muscle.

### Cognitive Demands Are Reduced When Performing Robot-Assisted Minimally Invasive Surgery

There is limited evidence on the cognitive fatigue experienced by surgeons performing MIS. The data that does exist predominantly involves subjective assessments using validated questionnaires. Indeed, to the best of the authors’ knowledge, no study has observed changes in brain activity during MIS utilising tools like electroencephalography (EEG), which could objectively directly quantify cognitive fatigue in surgeons.

A small number of studies have used indirect objective measures of cognitive fatigue, such as heart rate measures. Hubert and colleagues observed that heart rate parameters indexed greater cognitive demands in surgeons performing laparoscopic procedures. Interestingly, this was in contrast to their subjective data, which did not indicate a difference in cognitive fatigue between RALS and LS. Van der Schatte Olivier et al. also utilised heart rate parameters to index cognitive fatigue and again, and observed greater cognitive fatigue during LS versus RALS.

On the topic of cognitive fatigue, it should be noted that the differing demands of the surgical environments associated with LS and RALS may also contribute to greater cognitive fatigue in LS. When performing RALS, surgeons are sat comfortably, mostly unscrubbed. In contrast during LS, surgeons remain standing, wearing additional personal protective equipment (PPE). Work in emergency surgery has shown that surgeons perceive PPE to reduce comfort, increase fatigue and reduce overall surgical performance.^47^ The added musculoskeletal demands of LS, requiring muscle activation to remain standing, also place an increased cognitive burden on surgeons as the brain is required to maintain postural control.^48^

In addition, LS requires the need for surgeons to assume more uncomfortable positions to access difficult to reach structures or perform difficult tasks. In contrast, RALS provides a 3-dimensional view of the operating field, use endowristed instruments and the robot has a clutch mechanism which eliminates these challenges. Collectively, this all places an added cognitive demand when performing LS, which has been highlighted by the studies using perceived pain or discomfort scales, Borg CR-10 scale, NASA-TLX scores or Subjective Mental Effort Questionnaires. The elevated cognitive demand placed is as a result of dual tasking, controlling the movement of the body whilst trying to perform posture unrelated cognitive activity.^49^ Indeed, based on the limited data available, there was a consistent decrease in predominantly subjectively assessed cognitive load observed when procedures are performed using RALS.^29,33,34,36,38,39,41^ This is perhaps not surprising.

There is a *U*-shaped relationship between the efficacy of postural control and concurrent cognitive demands.^49^ The diminishing need to control posture in RALS removes the competition for cognitive resource, allowing surgeons to focus on the surgical task but ultimately reducing cognitive burden. Not a single study reported an increased cognitive demand with RALS. Whilst three studies using subjective measures of assessment reported no difference in cognitive load between LS and RALS,^35,37,40^ these findings are likely limited by the insensitivity of the methods employed.^50^ Collectively, data suggest that cognitive demand is greater in LS.

Finally, the surgical 1st assistants’ role differs markedly in RALS and LS. None of the studies have investigated how factors related to the assistants (e.g. experience, qualifications) affect surgeon fatigue. Additionally, demands on surgical assistants require further investigations because some studies observed less pain and discomfort compared to the primary operating surgeon in both RALS and LS^51^ and potentially increased cognitive fatigue.^52^

### Strengths and Limitations

To the authors’ knowledge, this is the first review that compares musculoskeletal demand and cognitive load in robot-assisted versus laparoscopic surgical techniques. Findings from the review indicate that RALS may be associated with less musculoskeletal and cognitive fatigue relative to LS.

An alternative hypothesis is that the increased muscle activity of the trapezius in RALS may be compensation for fatigue in the erector spinae that demonstrates lower muscle activation. Further work is required to determine whether these different patterns of recruitment are representative of compensation and present different areas of risk in RALS.

The previous studies included in the review that have compared musculoskeletal demand and cognitive load in RALS vs LS surgeries are variable in study type and quality, leading to heterogeneous data. These factors limit the conclusions that can be drawn. Whilst there are several benefits of RALS, these findings should be interpreted cautiously with the known limitations within the design of past studies. Indeed, confounders like surgeons’ handedness, BMI, diet, physical activity levels and experience were not controlled in most of these studies. Performing a (quantitative) meta-analysis was precluded by the significant heterogeneity in study designs, observed outcomes and study population.

### Future Research

Further research is required to understand how different postures can reduce musculoskeletal stress evidenced at the knee, ankle and foot in laparoscopic surgery. These challenges are specific to the laparoscopic domain but could benefit surgeon musculoskeletal health as particular postures are held for sustained durations. Further research is required to quantify cognitive fatigue in surgeons using objective measures such as EEG, which are less prone to the limitations of subjective assessment.^50,53,54^ This will provide a direct objective measurement of brain function, unlike indirect objective measurements based on heart rate or skin conductance. If changes in fatigue during surgery can be determined objectively, then the relationship to musculoskeletal fatigue should be investigated in tandem to determine if cognitive fatigue is causative of musculoskeletal fatigue, caused by a reduction in central drive. These results could form the evidence base for future designs of robotic consoles with improved ergonomic characteristics.

Further research is required to investigate the effect of procedure times on fatigue. Most of the studies in the review involved simulated fundamentals in laparoscopy skills (FLS) which require short amounts of time to complete, making any meaningful conclusions on the effect of time impossible.

Lastly, there is the potential to incorporate sensor systems that could aid the detection and monitoring of cognitive fatigue in surgeons to protect both surgeon musculoskeletal health and patient’s surgical outcomes.

## Supplementary Information

Below is the link to the electronic supplementary material.

Supplementary file1 (DOCX 21 KB)

## Declarations

### Registration

This review was registered in PROSPERO: International prospective register of systematic reviews and was assigned a registry iD CRD42020184881. This can be accessed using the link below and includes all associated changes made. https://www.crd.york.ac.uk/prospero/display_record.php?ID=CRD42020184881
