# Supplementary material for: Should All Minimal Access Surgery Be Robot-Assisted? A Systematic Review into the Musculoskeletal and Cognitive Demands of Laparoscopic and Robot-Assisted Laparoscopic Surgery
Source: J Gastrointest Surg. 2022 Apr 14;26(7):1520–30. doi: 10.1007/s11605-022-05319-8 (PMC9296389; doi:10.1007/s11605-022-05319-8)
Supplement: Supplementary file 1 — Supplementary file1 (DOCX 21 KB) [file 11605_2022_5319_MOESM1_ESM.docx]

**Supplemental 1: Medline Search Strategy**

1. exp SURGEONS/
2. exp "MUSCULOSKELETAL PHYSIOLOGICAL PHENOMENA"/
3. ("Laparoscopic surgery")
4. (robot-assisted)
5. ("robot assisted laparoscopic surgery")
6. (3 AND 4)
7. (1 AND 2 AND 7)
8. (posture)
9. ("muscle strain")
10. (9 OR 10)
11. (1 AND 3 AND 11)
12. (2 AND 3)
13. (1 AND 6)
14. (3 AND 11)
15. ("traditional laparoscopic surgery")
16. (ergonomics)
17. ("lower limb stress")
18. ("musculoskeletal disorder")
19. ("musculoskeletal disorders")
20. (19 OR 20)
21. (biomechanical)
22. ("physical burden")
23. ("physical discomfort")
24. ("laparoscopic procedures")
25. ("muscular activity")
26. (r-lap)
27. (c-lap)
28. (RAL)
29. (LS)
30. (RALS)
31. (27 OR 29 OR 31)
32. (28 OR 30)
33. (22 OR 23 OR 24 OR 26)
34. (3 OR 25)
35. (SURGEON)
36. (34 AND 35 AND 36)
37. (3 OR 6 OR 16 OR 27 OR 28 OR 29 OR 30 OR 31)
38. (34 AND 36 AND 38)
39. ERGONOMICS/
40. LAPAROSCOPY/
41. "MUSCLE CONTRACTION -- PHYSIOLOGY"/
42. "MUSCLE, SKELETAL -- PHYSIOLOGY"/
43. "ROBOTIC SURGICAL PROCEDURES"/
44. "STRESS, PHYSIOLOGICAL -- PHYSIOLOGY"/
45. "CHOLECYSTECTOMY, LAPAROSCOPIC -- METHODS"/
46. "OCCUPATIONAL DISEASES -- DIAGNOSIS"/
47. "ROBOTIC SURGICAL PROCEDURES -- METHODS"/
48. "BIOMECHANICAL PHENOMENA"/
49. "POSTURE -- PHYSIOLOGY"/
50. PHYSICIANS/
51. "BIOMECHANICAL PHENOMENA"/
52. "LAPAROSCOPY -- METHODS"
53. "OCCUPATIONAL DISEASES -- PREVENTION & CONTROL"
54. "OCCUPATIONAL DISEASES -- PHYSIOPATHOLOGY"/
55. "SURGICAL PROCEDURES, OPERATIVE -- METHODS"/
56. "PHYSICAL ENDURANCE -- PHYSIOLOGY"/
57. (41 AND 44)
58. (42 OR 43 OR 45 OR 47 OR 49 OR 50 OR 52 OR 56 OR 59)
59. (41 AND 51 AND 61)
60. (1 AND 41 AND 61)
61. (40 AND 41 AND 44)
62. (41 AND 61)
